# Comparative Analysis of Surgical and Endovascular Approaches for Isolated Aortic Coarctation Repair across Age Groups: Outcomes and Long-Term Efficacy

**DOI:** 10.3390/jcm13195814

**Published:** 2024-09-28

**Authors:** Nur Dikmen, Evren Ozcinar, Zeynep Eyileten, Ali Ihsan Hasde, Levent Yazicioglu, Bulent Kaya, Adnan Uysalel

**Affiliations:** Faculty of Medicine, Ankara University, 06100 Ankara, Türkiye; nurdikmen@yahoo.com (N.D.); zeyileten@gmail.com (Z.E.); ahasde@gmail.com (A.I.H.); leventyazicioglu@hotmail.com (L.Y.); bulentkaya@gmail.com (B.K.); uysalel@gmail.com (A.U.)

**Keywords:** aortic coarctation, aortic stenting, arterial hypertension, aortic re-coarctation

## Abstract

**Background:** Aortic coarctation, a condition characterized by localized narrowing of the aorta, can be managed with either surgical or endovascular techniques. This study aims to compare these approaches concerning long-term outcomes, particularly re-coarctation rates and late arterial hypertension. **Methods:** We retrospectively analyzed data from patients with native, isolated aortic coarctation treated by surgical or endovascular methods between 2015 and 2024. Clinical and demographic data were collected from electronic health records. Blood pressure was measured using oscillometric devices, and transthoracic echocardiography (TTE) was performed by an experienced sonographer. The primary endpoint was to identify which treatment predicted re-coarctation during follow-up, while the secondary endpoint assessed the incidence of late arterial hypertension. **Results:** Sixty-nine patients were included, with a mean age of 18.14 ± 8.18 years (median 16 years; range 8 to 37 years) and a median follow-up of 3 years (range 6 months to 8 years). Of these, 67 (97.1%) underwent elective repairs. Repair techniques included endovascular treatment (24.6%), surgical end-to-end anastomosis (47.8%), and surgical patchplasty (27.5%). The endovascular group was significantly older (29.82 ± 5.9 years vs. 14.33 ± 4.25 years, *p* = 0.056) and had shorter procedure durations and hospital stays. One-year freedom from reintervention was significantly higher in the surgical group (98.7%) compared to the endovascular group (88.23%) (*p* < 0.001). **Conclusions:** Both techniques effectively treat aortic coarctation, but surgical repair offers better long-term outcomes, while endovascular repair provides shorter recovery times. These findings should inform the choice of treatment modality based on patient-specific factors and clinical priorities.

## 1. Introduction

Coarctation of the aorta (CoA) is the sixth most common cardiovascular malformation, representing 5–8% of all congenital heart diseases, and is primarily diagnosed during infancy [1,2,3]. Since the pioneering surgical repair performed by Crawford in 1944 [4], surgical intervention has been the standard treatment for isolated CoA for over 50 years. Various surgical techniques have been developed, including subclavian flap repair, patch augmentation, and end-to-end anastomosis, which remains the most commonly employed method [5]. Despite the efficacy of surgical repair, untreated thoracic aortic coarctation can lead to severe complications, including early death due to hypertension and associated cardiovascular issues.

In recent years, balloon angioplasty and stenting have become significant alternatives, especially in older children and adults. These endovascular techniques emerged in the 1980s with balloon angioplasty, followed by stenting in the early 1990s, aiming to reduce surgery-related morbidity and acute complications [6,7]. Technological advancements and increased operator experience have enhanced the safety and success of endovascular CoA treatments. However, patients who undergo aortic stenting often continue to experience hypertension, even in the absence of residual obstruction [8].

It is important to note the increasing use of endovascular approaches in treating thoracic aortic conditions, with newer stent designs tailored to specific technical needs. Rizza et al. demonstrated the effectiveness of the Castor single-branched stent graft in Zone 2 thoracic endovascular aortic repair (TEVAR) procedures, showing promising preliminary outcomes with high technical success and low complication rates. Their findings highlight the growing trend of endovascular treatment as a viable alternative to traditional surgery, particularly for complex aortic pathologies [9].

Despite numerous studies comparing balloon angioplasty and surgical repair, there remains considerable debate regarding the optimal approach for managing both native and recurrent CoA [10,11]. Additionally, as highlighted in the work by Trimarchi et al., patients who undergo aortic coarctation repair, whether through surgical or endovascular means, are at risk of developing heart failure with preserved ejection fraction (HFpEF) in the long term, even after successful intervention. This underlines the importance of long-term cardiovascular follow-up and management to mitigate these risks [12]. This single-center, retrospective study aims to evaluate and compare the effectiveness of surgical and endovascular stenting techniques in terms of re-coarctation rates, as assessed by transthoracic echocardiography, and the incidence of late arterial hypertension, as measured by blood pressure monitoring, during the long-term follow-up of a cohort of young and adult patients.

## 2. Materials and Methods

We retrospectively reviewed the clinical information of all consecutive patients with native, isolated coarctation of aorta treated by surgical or endovascular techniques between 2015 and 2024. The study was conducted in accordance with the guidelines of the Declaration of Helsinki and was approved by the Research Ethics Board of Ankara University (approval date: 15 March 2024, protocol no: 2024/141). Written informed consent was obtained from all patients. Demographic and clinical data were extracted from the electronic health record system.

Demographic and clinical information included age, gender, and antihypertensive medication. All patients treated with aortic stenting performed the same percutaneous procedure with implantation of The BeGraft Aortic Stent Graft System (Bentley, Hechingen, Germany). Blood pressure (BP) was measured during clinical examinations using an oscillometric device on the right arm in the seated position. BP measurements were taken at rest, in accordance with pediatric guidelines recommendations [13]. Additionally, BP was measured in the supine position at both the right arm and right leg to assess any arm-to-leg gradient, defined as significant when ≥20 mmHg.

Transthoracic echocardiography (TTE) was performed on all patients by an experienced sonographer using standardized techniques.

The primary endpoint was to identify which treatment technique (surgical versus percutaneous stenting) predicted re-coarctation during long-term follow-up. The secondary endpoint was to evaluate the incidence of late arterial hypertension after different types of aortic repair.

In order to ensure effective long-term monitoring of patients postoperatively, we used a structured follow-up strategy that included initial visits at 1 month, followed by subsequent evaluations at 6 months, 1 year, and annually thereafter. During these visits, imaging modalities such as MRI were employed to assess vessel integrity, while CT scans were utilized to evaluate for potential complications, including re-coarctation or aneurysm formation. This comprehensive approach aimed to provide timely interventions and tailored care based on individual patient needs, ultimately improving long-term outcomes.

### 2.1. Techniques of Surgical Treatment

**Figure 1:**
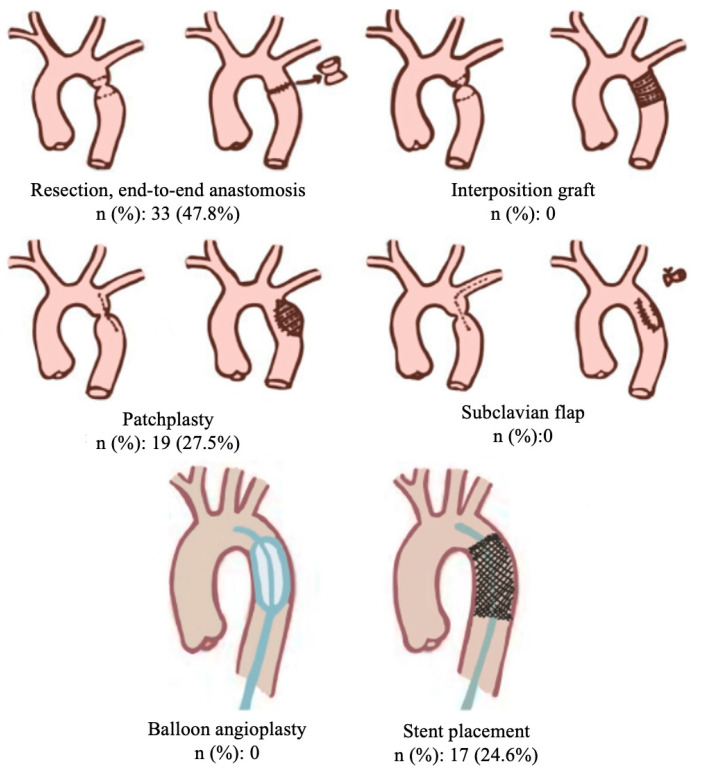
Schematic illustration of various surgical and endovascular techniques used in the repair of aortic coarctation. The top row shows surgical techniques: Resection with end-to-end anastomosis, interposition graft, patchplasty, and subclavian flap repair. The bottom row demonstrates endovascular approaches: balloon angioplasty and stent placement.

The surgical management of aortic coarctation, a condition characterized by a localized narrowing of the aorta, involves several advanced techniques aimed at restoring normal aortic diameter and optimizing hemodynamics. The primary surgical approach is the resection of the coarctation segment with end-to-end anastomosis, where the narrowed segment of the aorta is excised, and the healthy ends are directly sutured together. Another technique, known as the subclavian flap aortoplasty, involves mobilizing a segment of the subclavian artery to augment the aortic wall at the site of coarctation, thereby widening the lumen. This is not a technique that we preferred for our patient population in this study. Additionally, the use of prosthetic grafts has become increasingly prevalent, where a synthetic conduit is interposed between the proximal and distal segments of the aorta to bridge the coarcted region. Each technique is selected based on factors such as the patient’s age, the severity of the coarctation, and associated anatomical considerations, with the overarching goal of restoring normal aortic function and mitigating the risk of long-term complications. Patchplasty involves the use of a patch to widen the narrowed segment of the vessel, providing benefits such as reduced tension on the anastomosis and improved blood flow. However, it may carry risks of patch-related complications. End-to-end anastomosis, on the other hand, directly connects the two ends of the vessel, offering a more straightforward approach with potentially lower complication rates, but it may not be suitable in cases of significant vessel discrepancy (Figure 1).

### 2.2. Techniques of Endovascular Repair

Endovascular interventions are typically conducted under general anesthesia or deep sedation due to the significant discomfort associated with coarctation dilation. The use of an endovascular suite is preferred for optimal imaging, and the involvement of a highly skilled team is crucial in mitigating the risks associated with these often medically complex cases.

While transfemoral access remains the standard approach, alternative methods such as contralateral transfemoral, transbrachial, or transeptal (via the right atrium) catheterization may be employed under specific conditions to enable imaging of the proximal aorta during the procedure. Typically, angiography and pressure gradients across the lesion are assessed both before and after treatment. In cases where aortic dimensions have not been pre-determined, a calibrated pigtail catheter may be utilized to measure these. Additionally, the increasing use of percutaneous closure devices has enhanced the feasibility and practicality of endovascular techniques (Figure 1).

### 2.3. Statistical Analysis

All continuous variables were assessed for normality with the Shapiro–Wilk test and by visual inspection of histograms. Variables following a normal distribution were expressed as means and standard deviations, while non-parametric variables were reported as medians with interquartile ranges. Differences between groups were tested using the Kruskall–Wallis test and the Mann–Whitney U test, as appropriate. Comparisons between groups for ICU stay and hospital stay were analyzed using the Mann–Whitney U test for non-parametric data, given the potential for non-normal distribution. Categorical variables were expressed as percentages and analyzed using the chi-squared test. A *p*-value of less than 0.05 was considered statistically significant. All tests were two-tailed, and statistical analyses were performed using SPSS software (Version 20.0, Chicago, IL, USA).

## 3. Results

A total of 69 patients were included in the study, of which 14 (20.3%) were male. The mean age of the cohort was 18.14 ± 8.18 years. The median follow-up duration was 3 (ranged between 1 to 8) years. Hypertension (HTN) was present in 29 patients (42%), and 3 patients (43%) had congestive heart failure (CHF). Among the cohort, 2 patients (29%) had other congenital heart defects (CHD), and 29 patients (42%) were on antihypertensive medications. Specifically, 16 (23.2%) were prescribed beta-blockers (BBs), 9 (13%) were on ACE inhibitors/ARBs, and 4 (5.8%) were on diuretics. Recurrent aortic coarctation (ACoA) was observed in eight patients (11.6%).

**Graph 1:**
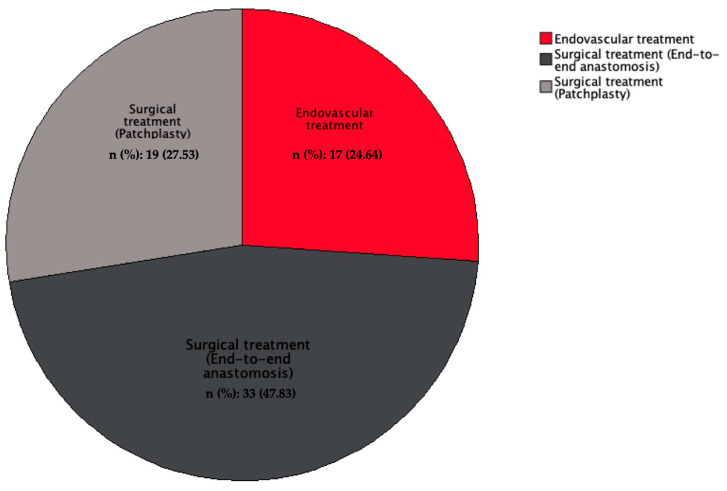
Patient groups of endovascular and different surgical treatment techniques.

**Figure 2:**
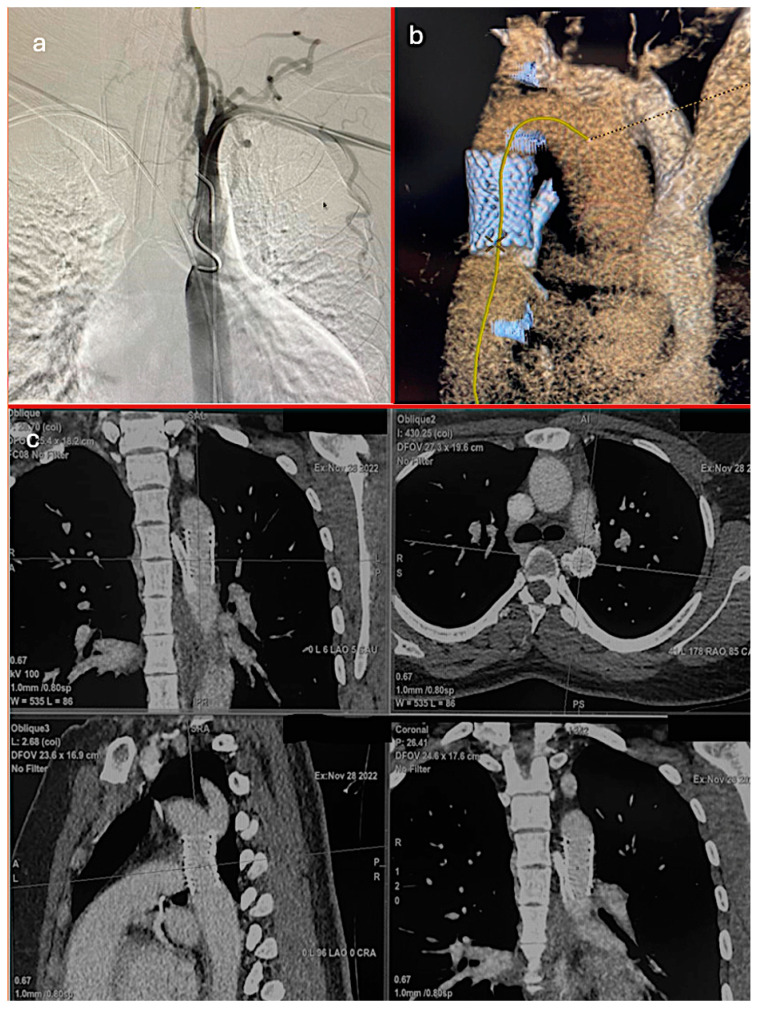
(**a**) Preoperative angiography of coarctation, (**b**) postoperative 3D CT imaging of aortic graft and chimney ARSA stent, (**c**) postoperative CT imaging of aortic graft and chimney ARSA stent.

Regarding the timing of the interventions, 67 patients (97.1%) underwent elective repairs, and 2 (2.9%) required emergent procedures. The repair techniques included endovascular treatment in 17 patients (24.6%), surgical end-to-end anastomosis in 33 patients (47.8%), and surgical patchplasty in 19 patients (27.5%). Figure 1 provides a detailed visualization of the aortic coarctation case in one of our patients. The postoperative CT scan confirmed the proper positioning of the aortic graft and chimney ARSA stent, illustrating the favorable outcome of the surgical intervention (Table 1, Graph 1, Figure 2).

Table 1 provides a detailed summary of the baseline demographic and clinical characteristics of the study population, consisting of 69 patients. Variables include gender distribution, age, prevalence of hypertension, heart conditions, and the type and timing of aortic coarctation repair. Additionally, the use of antihypertensive medications is outlined.

When comparing the clinical variables between the surgical and endovascular treatment groups, patients in the endovascular group were significantly older at the time of repair (29.82 ± 5.9 years) compared to the surgical group (14.33 ± 4.25 years) (*p* = 0.056). The percentage of males was significantly higher in the endovascular group (47.5%) compared to the surgical group (11.5%) (*p* < 0.001). There was no significant difference in the use of antihypertensive medications between the groups, with 73.7% of the surgical group and 88.23% of the endovascular group on anti-HTN therapy (*p* = 0.746).

The duration of the procedure was significantly shorter for the endovascular group (34.06 ± 8.14 min) compared to the surgical group (85.21 ± 16.57 min) (*p* = 0.005). Postoperative intensive care unit (ICU) stay was markedly reduced in the endovascular group (1.53 ± 0.62 h) compared to the surgical group (33.27 ± 11.32 h) (*p* < 0.001). Similarly, the postoperative hospital stay was shorter for the endovascular group (2.29 ± 0.77 days) compared to the surgical group (6.38 ± 0.99 days) (*p* < 0.001).

**Graph 2:**
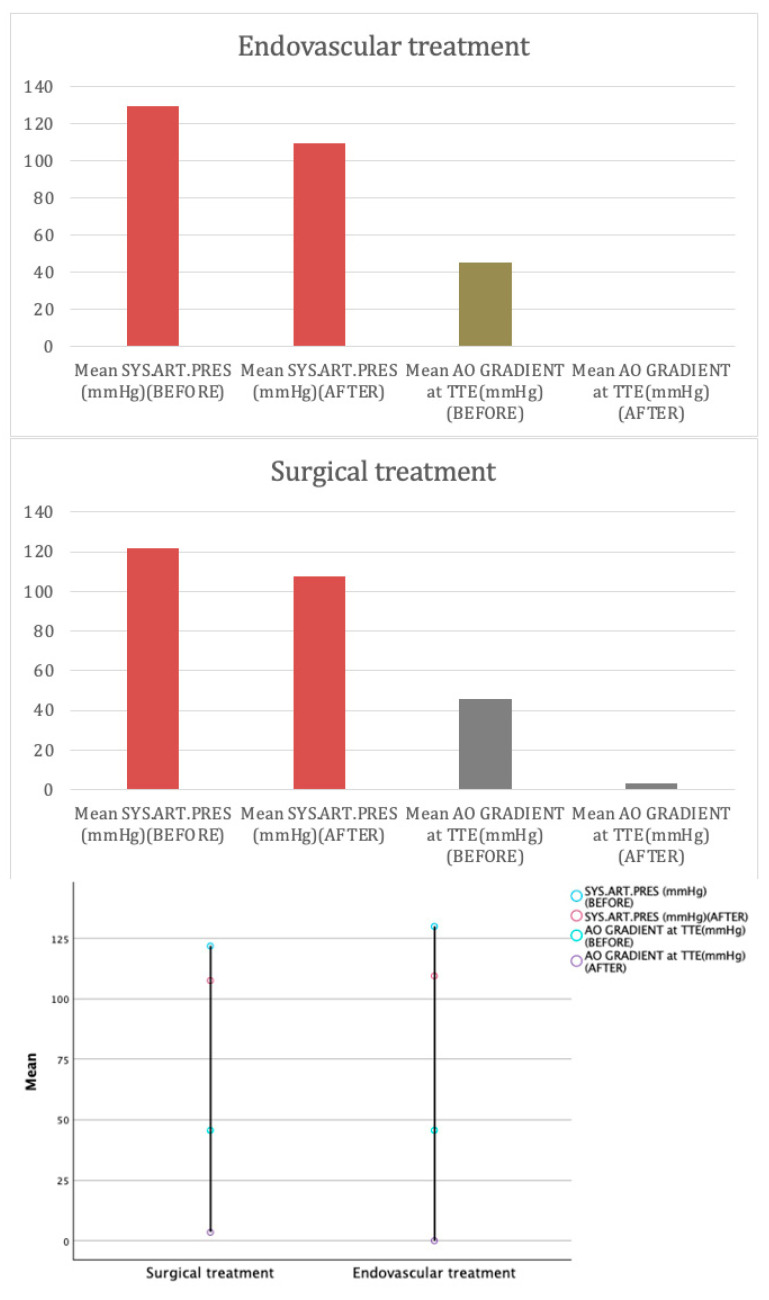
The changes in mean systolic blood pressures and aortic gradients before and after surgical and endovascular techniques. This graph illustrates the changes in systolic arterial pressure and the pre- and post-treatment aortic gradients before and after treatment in both the endovascular and surgical groups. A significant reduction in pressure was observed post treatment for both groups, with slightly greater reductions in the endovascular group. The endovascular group achieved complete resolution of the aortic gradient (0 mmHg), while the surgical group showed a partial reduction with residual gradients observed post surgery.

**Figure 3:**
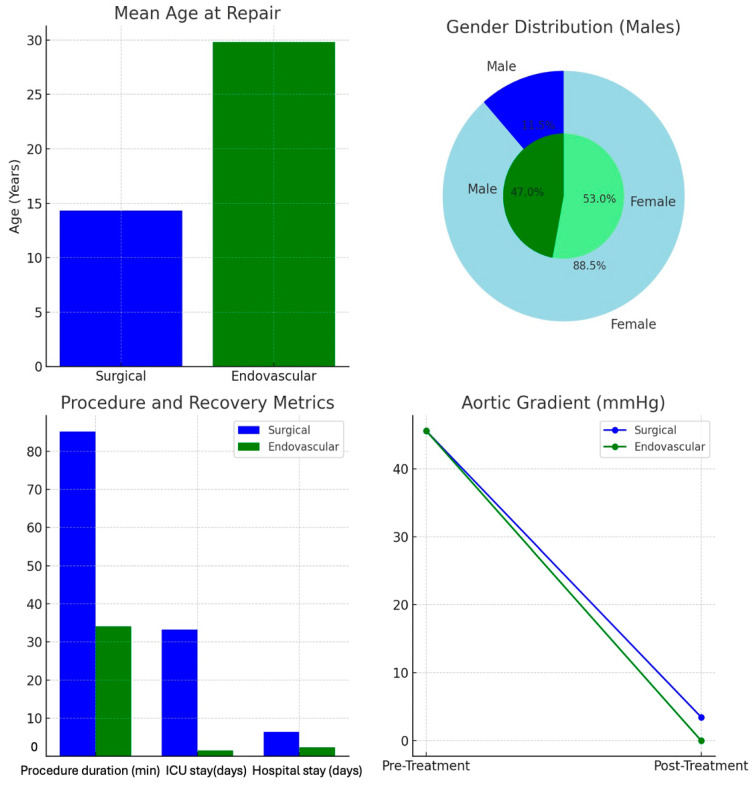
Comparison of demographic and clinical outcomes between surgical and endovascular treatments for aortic coarctation. This figure compares the outcomes of surgical versus endovascular treatments for aortic coarctation across four key metrics: patient demographics, procedure duration, recovery time, and aortic gradient reduction.

Regarding blood transfusions, the median transfusion volume was significantly higher in the surgical group, where no transfusions were required in the endovascular group (*p* < 0.001). There was no significant difference between groups in terms of pre-treatment systolic blood pressure (SBP) or pre-treatment aortic gradients (*p* > 0.05). Post treatment, the aortic gradient on transthoracic echocardiography (TTE) was significantly lower in the endovascular group, with a mean gradient of 0 mmHg compared to 34.6 ± 1.56 mmHg in the surgical group (*p* < 0.001) (Graph 2 and Figure 3).

In the top-left chart, the mean age at repair was significantly higher in the endovascular group (29.82 years) compared to the surgical group (14.33 years), indicating that endovascular treatments are more commonly performed in older patients. The top-right chart shows gender distribution, highlighting a greater proportion of males in the endovascular group (47%) compared to the surgical group (11.5%). This suggests a gender disparity, with males more frequently undergoing endovascular procedures. The bottom-left bar chart compares procedure duration, ICU stay, and hospital stay. The endovascular group showed shorter procedure durations (34.06 min vs. 85.21 min), ICU stays (1.53 h vs. 33.27 h), and hospital stays (2.29 days vs. 6.38 days), highlighting the less invasive and quicker recovery nature of endovascular treatment. Finally, the bottom-right line graph illustrates aortic gradient reduction. Both groups showed a significant decrease in aortic gradient post treatment, with the endovascular group achieving complete resolution (0 mmHg), and the surgical group showing a residual gradient (3.46 mmHg), suggesting superior immediate hemodynamic outcomes with endovascular procedures.

This overall comparison indicates that while endovascular procedures offer quicker recovery and excellent immediate outcomes, they are generally performed on older patients and may have long-term considerations.

**Graph 3:**
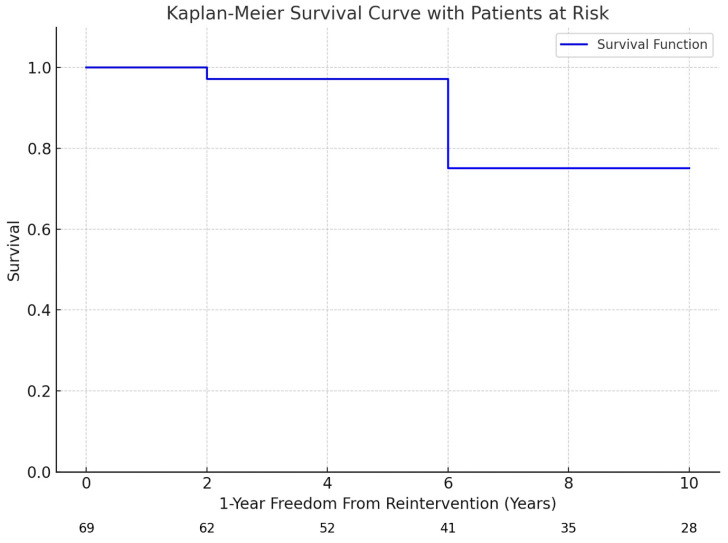
Kaplan–Meier survival curve with 1-year freedom from reintervention and patients at risk over time. The Kaplan–Meier survival curve shows the probability of patients remaining free from reintervention over time. The blue line represents the survival function, with the x-axis indicating the follow-up period in years, and the y-axis shows the probability of survival (i.e., the percentage of patients who did not require reintervention at each time point). Below the x-axis, the numbers reflect the patients at risk at specific time intervals (0, 2, 4, 6, 8, and 10 years). At the beginning of the follow-up period (year 0), 69 patients were at risk, with the number gradually decreasing over time due to reinterventions or censored data. The absence of censored events in this plot means all patients were included in the follow-up for the duration shown. This graph provides insight into the long-term durability of the treatment in preventing reinterventions.

**Figure 4:**
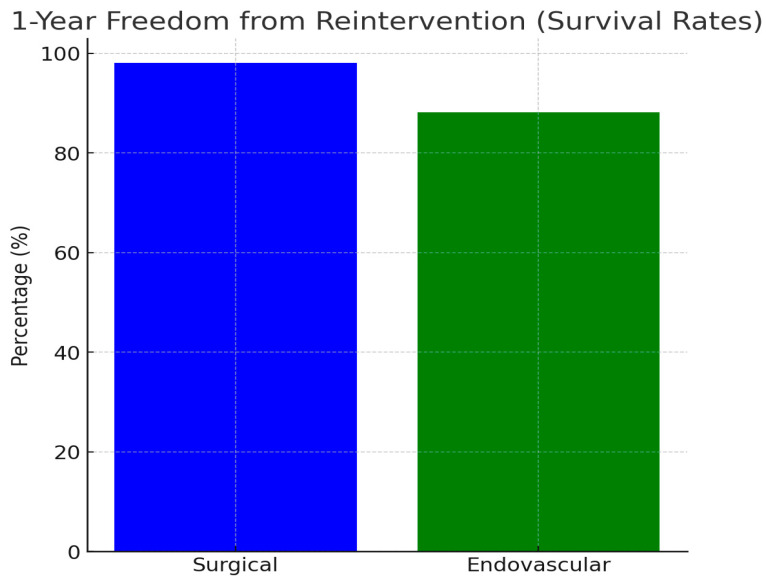
Comparison of 1-year freedom from reintervention between surgical and endovascular treatments. This figure illustrates the comparative outcomes between surgical and endovascular treatments for patients undergoing aortic coarctation repair, specifically focusing on the 1-year freedom from reintervention, which serves as a proxy for treatment durability and success over time. Surgical treatment exhibited a significantly higher 1-year freedom from reintervention rate, reaching 98.07%, compared to 88.23% for the endovascular treatment group. (The blue bar represents the surgical treatment, and the green bar represents the endovascular treatment. The y-axis shows the percentage of patients free from reintervention, while the x-axis lists the two types of procedures compared. This figure highlights the critical consideration of treatment durability, offering insight into long-term planning and patient care following aortic coarctation repair).

Early reintervention was required in one patient (19.2%) in the surgical group, due to insufficient reduction of the aortic gradient, via the technique of end-to-end anastomosis, observed during intraoperative measurements, patchplasty was performed. In addition, early reintervention was required for two patients (11.7%) in the endovascular group. During intraoperative angiography, insufficient stent dilatation of the coarctation site was observed, leading to the decision to repeat balloon angioplasty. (*p* < 0.001). Given the higher rates of re-coarctation observed in the endovascular group, it is essential to examine the contributing factors that may predispose these patients to complications. Key considerations include vessel elasticity, which affects the ability of the stent to maintain proper dilation; the precision of stent placement techniques, which can impact the stent’s effectiveness; and specific anatomical variations that may influence the overall success of the endovascular repair. Understanding these factors is crucial, as they may elucidate the need for balloon angioplasty following stenting due to recoil rates. No mortality or major complications were observed in either group within the 30-day postoperative period. One-year freedom from reintervention was significantly higher in the surgical group (98.7%) compared to the endovascular group (88.23%) (*p* < 0.001). The aforementioned reinterventions were identified during the intraoperative period and were addressed immediately during the surgery (Table 2, Graph 3, Figure 4).

Table 2 compares the clinical variables and outcomes between the surgical and endovascular treatment groups. It includes age, sex distribution, medication use, procedural duration, ICU stay, hospital stay, transfusion requirements, and pre- and post-treatment hemodynamic data. The table also reports the rates of early reintervention, complications, and 1-year reintervention-free survival between the two groups.

In Table 3, we present the outcomes of patients stratified by age, specifically comparing those under and over 18 years. Given that the patient numbers were not evenly distributed between the groups, conducting this analysis in a larger cohort with equal distribution in future studies would significantly contribute to the literature. Additionally, the early outcomes observed in this study provide valuable insights, serving as a foundation for further research into age-specific treatment effectiveness (Table 3).

This table compares outcomes of patients undergoing treatment for coarctation, categorized by age group: ≥18 years (*n* = 29) and <18 years (*n* = 40). Outcomes assessed include rates of recurrent coarctation and early reintervention, as well as systolic arterial pressure values before and after treatment and aortic gradient measured by transthoracic echocardiography (TTE). *P* values are included to indicate statistical significance. A significant difference in the aortic gradient after treatment was observed between the age groups (*p* = 0.006), while no statistically significant differences were found in other parameters.

## 4. Discussion

Coarctation of the aorta (CoA) is a congenital narrowing of the aorta, most frequently occurring at the level of the ductus arteriosus or ligamentum arteriosum, typically proximal to the thoracic aorta. It is the third most prevalent congenital heart defect, with an occurrence rate ranging from 20 to 60 cases per 100,000 live births, constituting from 5 to 8% of all congenital heart diseases [14]. This condition is primarily diagnosed in infancy and early childhood. Surgical repair has long been the standard treatment for neonates, infants, and young children [15].

The morphological spectrum of aortic coarctation exhibits significant variability, ranging from discrete stenosis located distal to the left subclavian artery to more severe manifestations, such as hypoplasia of the aortic arch and isthmus. Arch hypoplasia is observed in approximately 25%–50% of cases. In certain instances, aortic coarctation may present as a long, tubular stenosis of the descending thoracic aorta, which can complicate diagnosis and management [16].

Coarctation can be classified as either a primary (native) condition or as a recurrent phenomenon following previous surgical intervention. Although management strategies for native and recurrent coarctation share similarities, the underlying pathophysiological processes differ, which can affect both treatment approaches and outcomes.

Untreated coarctation of the aorta is associated with considerable morbidity and early mortality due to a range of complications, including hypertension, congestive heart failure, myocardial infarction, stroke, aortic rupture, and infective endocarditis. The mean age of death for individuals with untreated coarctation is estimated to be between 33 and 35 years, with approximately 90% of patients succumbing by the sixth decade of life [13].

Coarctation of the aorta is predominantly diagnosed in children, with approximately one-third of cases classified as critical coarctation during infancy [17]. In these situations, severe stenosis becomes apparent upon the closure of the ductus arteriosus, often leading to cardiac failure due to aortic obstruction. If the ductus remains patent, critical stenosis may go undetected. In cases of less severe narrowing, collateral circulation can develop around the coarctation, potentially delaying diagnosis until later in childhood. Approximately 20% of patients may not present until adulthood, with incidental hypertension being the usual clinical manifestation. However, some individuals may present with severe complications, including heart failure, aortic rupture or dissection, infective endocarditis, or stroke [18].

### 4.1. Treatment

The timing of repair is a critical factor influencing outcomes, particularly in younger patients. Early surgical intervention for native coarctation of the aorta is associated with a significantly reduced risk of developing late hypertension and is linked to improved survival rates. Timely corrective surgery can mitigate long-term complications and enhance overall outcomes for affected individuals. However, early repair is also linked with a higher risk of re-coarctation [19]. Although the risk of re-coarctation is lower with delayed intervention, the likelihood of developing late hypertension and other long-term complications increases [20]. Despite presenting at a later age, both surgical and endovascular interventions have been shown to improve survival rates and hypertension outcomes in patients with aorto-occlusive disease [21,22].

The treatment of aortic coarctation has evolved considerably since Crafoord’s pioneering surgical repair in 1944 [23]. Open surgical techniques have remained the primary mode of intervention since then, leading to gradual improvements in morbidity and mortality rates over the years. Contemporary surgical methods have produced generally excellent outcomes, reflecting advancements in both technique and postoperative care [24].

### 4.2. Surgical Treatment

Early surgical outcomes were marked by high perioperative mortality and morbidity, especially among neonates. However, advancements in surgical techniques and increased clinical experience over the past 10–15 years have substantially reduced the incidence of adverse events [25,26]. In the most extensive published series to date, perioperative mortality rates averaged 2.6%, with peak rates observed in patients younger than 1 year (6.5%) and those older than 30 years (4.5%). Aneurysm development post coarctation repair can happen at both early and late stages; however, inconsistencies in definitions complicate the evaluation between surgical and endovascular methods [27,28].

Paraparesis due to spinal blood flow interruption during open repair occurs in between 0.3% and 2.4% of cases but is usually transient. Paradoxical hypertension is frequently reported after surgery, occurring in up to 76% of cases. Additionally, post-coarctation syndrome, associated with the reperfusion of abdominal viscera, is relatively common in certain surgical series [26,29]. In spite of these difficulties, endovascular methods present a less invasive option compared to conventional open surgery, often leading to reduced hospital stays and achieving widespread recognition. Nevertheless, the optimal management strategy remains debated, with limited robust comparative data between surgical and endovascular therapies [16]. Variations in patient populations, characteristics, and outcome measures, combined with evolving techniques, complicate comparisons. To date, the sole two randomized prospective studies performed so far were modest in scale and underpowered, thereby limiting their potential to deliver conclusive results [30].

### 4.3. Endovascular Treatment

The first reported angioplasty for coarctation occurred in 1981 [31]. Initially limited to cases of re-coarctation, its application has since broadened to encompass native coarctation, demonstrating comparable primary outcomes [32]. Angioplasty has been applied across all age groups, with longer-term data now available. Early reports indicated a high complication rate, including restenosis and aneurysm formation, which improved with advancements in technique. The initial application of stents for aortic coarctation was documented in 1991, and technological advancements have enhanced endovascular repair by enabling smaller delivery systems and a broader array of devices [33]. Stenting has become a prevalent approach for older children and adults, demonstrating cost-effectiveness compared to surgical intervention [34,35].

Angioplasty facilitates luminal expansion by dilating and rupturing the intimal layer of the coarctation segment, with varying degrees of penetration into the media of the vessel wall. The selection of balloon size is essential to reduce elastic recoil and enhance luminal gain while avoiding undue injury to a potentially weakened vessel wall. Particular care is recommended for patients over 50 years of age, especially if the aorta is calcified, as degenerative changes may further compromise the strength of the arterial wall [36].

Underdistension can lead to residual stenosis, whereas overdistension poses risks of aortic dissection, rupture, or aneurysm formation. Stenting reduces trauma to the vessel wall by preventing overdistension, minimizing elastic recoil, and stabilizing small dissections with the struts of the stent. While the neointimal response may be more significant with stenting, this is likely balanced by the greater initial luminal gain obtained [37].

Predictors of unfavorable initial outcomes include nondiscrete lesions and inadequate operator experience. The higher primary success rate of stenting for native coarctation compared to re-coarctation may be attributed to the increased resistance of secondary stenosis to balloon treatment, primarily due to the presence of scar tissue [38]. Stent placement for native coarctation demonstrates outstanding primary outcomes in over 95% of patients, with a significantly improved mean end gradient compared to angioplasty alone. Stenting is also more effective for nondiscrete coarctation, and the results for recurrent coarctation are similar to those for native stenosis [29].

There have been few reported deaths following angioplasty for native coarctation, with most series indicating no periprocedural mortality. The mortality rates for angioplasty of re-coarctation are similar to those for native lesions, ranging from 0.7% to 1.1%. Scar tissue from prior repairs may increase the resistance of the re-coarcted segment to rupture, aneurysm development, and dissection compared to native coarctation. The frequency of aneurysms after angioplasty for native coarctation has been a subject of discussion, with early reports suggesting an incidence as high as 50%. A small, randomized trial that compared angioplasty and surgical intervention in children found a 20% risk of aneurysm in the short term and a 30% risk in the long term following angioplasty, whereas no risk was observed after surgical repair [39].

Recurrent narrowing is rare but can occur with transverse stent fractures. There is a theoretical risk of complications such as dissection, aneurysm, or perforation due to mural trauma. Stent fractures may require additional stenting or grafting. Stent migration, often due to slippage or balloon rupture, is uncommon, while true late migration is extremely rare. However, circumferential stent fractures might lead to distal embolization of stent fragments [40,41].

### 4.4. Follow-Up

A clinical review is recommended between 4 and 6 weeks post procedure, with long-term follow-up necessary due to risks of restenosis, late-onset hypertension, and aneurysm formation. Normotensive patients at initial follow-up may benefit from a trial discontinuation of antihypertensive medication, though many advocate for ongoing exercise or ambulatory blood pressure monitoring due to the potential for undetected hypertension [20].

Multidetector computed tomography angiography (MDCTA) and magnetic resonance imaging (MRI) are preferred for follow-up in older children and adults, while catheter angiography remains useful for evaluating potential restenosis. Restenosis rates following surgical repair of native coarctation range from 1.7% to 50%, though modern techniques have reduced this risk. Factors such as younger age at repair and aortic arch hypoplasia increase the likelihood of restenosis, with recurrent stenosis rates after repeat surgery between 7% and 30% [42].

For angioplasty, recurrent coarctation is noted, particularly in neonates, but tends to decrease with age, with rates from 0% to 15% in adults. Angioplasty remains a valuable palliative option, with generally favorable outcomes even in young patients. Stenting, in comparison, shows lower re-coarctation rates due to reduced elastic recoil, although mild intimal hyperplasia is common. The greater initial luminal gain from stenting helps mitigate potential restenosis [43].

Postoperative imaging results, including CT scans and angiography, are essential for guiding clinical decision-making and evaluating long-term patient outcomes. These imaging modalities provide critical insights into the vascular architecture and any potential complications, such as re-coarctation or aneurysm formation. By assessing the imaging findings, clinicians can make informed decisions regarding the necessity for further interventions, adjustments in medication management, or the timing of follow-up evaluations, ultimately leading to personalized care strategies that improve patient outcomes [41].

### 4.5. Hypertension

Hypertension is a critical outcome following coarctation repair due to its correlation with premature death risk. The prevalence of late hypertension varies widely, from 7% to 75%, with up to 45% of these patients showing no recurrent stenosis. Our study’s results indicate that 42% of patients experienced hypertension, aligning with the wide variability in hypertension prevalence reported in the literature. Early surgical repair is associated with a lower risk of late hypertension and improved long-term survival. Even among those repaired in infancy, 21% may develop high blood pressure from 10 to 12 years later. The optimal age for repair is around 1.5 years, but advances may shift this earlier. Long-term, 30–40 years post-surgery, from 32% to 34% of patients may remain normotensive [44,45,46].

The prevalence of hypertension following endovascular repair is less well defined, but early improvements are observed in up to 95% of patients post-stenting. However, hypertension persists in up to one-third of adults after treatment, though between 50% and 75% may see improved blood pressure control or discontinue medication in the intermediate term [19].

Endovascular treatments, including balloon angioplasty and stenting, have emerged as promising alternatives for both primary and secondary coarctation repair. While stenting offers superior immediate relief of pressure gradients and a lower risk of restenosis and aneurysm formation compared to balloon angioplasty alone, surgical repair remains linked to fewer acute complications and better long-term hemodynamic outcomes. Recent studies have suggested that surgical repair may be preferable for native coarctation in children, while stenting might be more suitable for re-coarctation, particularly in post-pubertal patients [47,48,49]. In our study, the cohort’s division between surgical (end-to-end anastomosis and patchplasty) and endovascular repair provides a basis for comparing outcomes across these methods. The results show that endovascular repairs were performed in significantly older patients (mean age 29.82 years) compared to those undergoing surgical repair (mean age 14.33 years) (*p* = 0.056). This age difference is consistent with the trend of reserving endovascular treatments for older patients or those with recurrent coarctation.

Animal studies have indicated that aortic stenting may cause endothelial dysfunction and elevated cardiovascular risk, with clinical data suggesting a greater need for hypertension therapy in stented patients. Thus, while both surgical and endovascular approaches have their merits, individualized treatment strategies should be considered based on patient characteristics and repair history [1,50].

Consistent with these findings, our data indicate that patients who received aortic stenting had a greater need for hypertension therapy compared to those who underwent surgical repair. Stenting may reduce aortic compliance and increase impedance to blood flow, leading to a loss of pulsatile energy, which could explain the lower pulse pressure values observed in patients with one-time surgical aortic repair compared to those with stent implantation. While the optional technique for isolated coarctation repair remains unresolved, our data suggest that surgical repair may still be the preferred option for native coarctation in the pediatric population. Conversely, for cases of re-coarctation, treatment strategies should be individualized. Post puberty, patients may present similar characteristics to adults, where stenting might be a more suitable option for re-coarctation correction.

### 4.6. Limitations

The primary limitation of our study is the heterogeneity among groups, particularly with respect to the age at first repair, which was higher in patients who underwent aortic stenting. This discrepancy arises because percutaneous treatments inherently require older and larger patients compared to surgical interventions, making age-matched groups impractical.

## 5. Conclusions

The field of endovascular management for coarctation is advancing. Currently, angioplasty plays a limited role in the management of severe coarctation in young children because of the high risk of subsequent re-coarctation, although it continues to be advantageous for addressing recurrent coarctation. Endovascular techniques have demonstrated safety and effectiveness in older children and adults. The placement of stents may provide specific benefits in cases of nondiscrete coarctation and is associated with low rates of re-coarctation.

## Figures and Tables

**Table 1:** Characteristics of the patient population.

Patients, *n*	69
Male, *n* (%)	14 (20.3)
Age, years (mean ± SD)	18.14 ± 8.18 (median:16, min–max: 8–37)
Hypertension, *n* (%)	29 (42)
CHF, *n* (%)	3 (4.3)
Other CHD, *n* (%)	2 (2.9)
HTN medications, *n* (%)	29 (42)
BBs, *n* (%)	16 (23.2)
ACEI/ARB, *n* (%)	9 (13)
Diuretics, *n* (%)	4 (5.8)
Recurrent ACoA, *n* (%)	8 (11.6)
Timing of repair	
Elective, *n* (%)	67 (97.1)
Emergent, *n* (%)	2 (2.9)
Repair techniques, *n* (%)	
Endovascular	17 (24.6)
Surgical, end-to-end anastomosis	33 (47.8)
Surgical, patchplasty	19 (27.5)

Abbreviations: SD: standard deviation; CHF: congestive heart failure; CHD: congenital heart defects; HTN: hypertension; ACoA: aortic coarctation.

**Table 2:** Clinical variables and outcomes of the two study groups.

	Surgical Treatment	Endovascular Treatment	*p* Value
Age at repair, years (mean ± SD)	14.33 ± 4.25	29.82 ± 5.9	0.056
Male, *n* (%)	6 (11,5)	8 (47.05)	<0.001
Anti HTN medication, *n* (%)	38 (73.07)	15 (88.23)	0.746
Procedure duration, min (mean ± SD)	85.21 ± 16.57	34.06 ± 8.14	0.005
PO ICU stay, hours (mean ± SD)	33.27 ±11.32	1.53 ± 0.62	<0.001
PO hospital stay, days (mean ± SD)	6.38 ± 0.99	2.29 ± 0.77	<0.001
Transfusion, ml (median, min-max)	0	100 (0–600)	<0.001
Pretreatment, mmHg (mean ± SD)	121.77 ± 12.27	129.82 ± 11.2	0.336
Post-treatment SBP, mmHg (mean ± SD)	107.5 ± 10.02	109.41 ± 8.81	0.398
Pretreatment Ao gradient at TTE, mmHg (mean ± SD)	45.58 ± 7.18	45.59 ± 7.68	0.808
Post-treatment Ao gradient at TTE, mmHg (mean ± SD)	3.46 ± 1.56	0	<0.001
Early reintervention, *n* (%)	1 (1.92)	2 (11.7)	<0.001
30-day mortality, *n* (%)	0 (0)	0 (0)	*
Complications, *n* (%)	0 (0)	0 (0)	*
1-year freedom from reintervention, *n* (%)	51 (98.07)	15 (88.23)	<0.001

Abbreviations: SD: standard deviation; HTN: hypertension; PO: postoperative; ICU: intensive care unit; SBP: systolic blood pressure; Ao: aorta; TTE: transthoracic echocardiography. (* There is no difference since both groups are zero).

**Table 3:** Analysis of outcomes in coarctation treatment by age group. Abbreviations: Sys: systolic; Art: arterial; Ao: aortic; TTE: transthoracic echocardiography) (p < 0.05 was considered statistically significant).

	Patients’ Age ≥ 18 (*n*: 29)	Patients’ Age < 18 (*n*: 40)	*p* Value
Recurrent Coarctation, *n*	6	2	0.10
Early reintervention, *n*	2	1	0.77
Sys.Art.Pressure (mmHg) (Before treatment)	124.62	123.12	0.34
Sys.Art.Pressure (mmHg) (After treatment)	107.41	108.37	0.69
Ao. Gradient at TTE (mmHg) (Before treatment)	45.17	45.87	0.74
Ao.Gradient at TTE (mmHg) (After treatment)	0.68	4.0	0.006

## Data Availability

The data presented in this study are available on request from the corresponding author due to privacy and ethical reasons.
